# Complete Chloroplast Genome Sequence of the Endemic and Medicinal Plant *Zingiber salarkhanii:* Comparative Analysis and Phylogenetic Relationships

**DOI:** 10.3390/biology15010014

**Published:** 2025-12-20

**Authors:** Mohammad Rashedul Islam, Dhafer A. Alzahrani, Enas J. Albokhari, Mohammad S. Alawfi, Arwa I. Alsubhi

**Affiliations:** 1 Department of Biological Sciences, Faculty of Sciences, King Abdulaziz University, Jeddah 21589, Saudi Arabia; dalzahrani@kau.edu.sa; 2 Department of Biology, Faculty of Sciences, Umm Al-Qura University, Makkah 24381, Saudi Arabia; ejbokhary@uqu.edu.sa; 3 Department of Biology, College of Sciences, King Khalid University, Abha 61421, Saudi Arabia; malawfi@kku.edu.sa; 4 Department of Biology, College of Science, Taibah University, Madinah 42353, Saudi Arabia; asubhia@taibahu.edu.sa

**Keywords:** *Zingiber salarkhanii*, endemic, Bangladesh, chloroplast (cp) genome, codon usage bias, SSRs, phylogenetic

## Abstract

*Zingiber salarkhanii* (Zingiberaceae family) is an endemic species of Bangladesh. It possesses biological effects, including analgesic, anxiolytic, cytotoxic, and antioxidant properties. Although genomic data on *Zingiber* is scarce, the entire chloroplast (cp) genome has been extensively used as a molecular marker to resolve phylogenetic issues. The genome size is 163,980 bp, and it has a standard quadripartite structure, with an average GC content of 36.91%. The genome contains 138 genes (113 unique), comprising 90 protein-coding genes, 79 unique genes, 48 noncoding genes (34 unique), 40 transfer RNAs (tRNAs), and eight ribosomal RNAs (rRNAs). Codon usage analysis of the cp revealed 14 high-frequency codons besides 18 optimal codons in this species. A repetitive study revealed 211 simple sequence repeats (SSRs), predominantly A/T mononucleotide repeats. Sequence alignment indicated that variable regions were primarily located in the single-copy regions. Sequence comparison showed that most variable regions were located within the single-copy regions, and nucleotide diversity (π = 0–0.11289) indicated overall low divergence with 11 mutation hotspots. Phylogenetic investigations using both coding sequences and complete cp genomes indicated that *Z. salarkhanii* is most closely related to the *Zingiber* genus. Phylogenetic investigations using both coding genes and complete cp genomes placed *Z. salarkhanii* within the core *Zingiber* lineage, revealing its closest relationship to *Z. recurvatum* rather than to the genus. It conducted an extensive analysis of many cp genomic characteristics for phylogenetic significance, including overall genome architecture, codon usage bias, repetitive sequences, inverted repeat borders, and phylogenetic reconstruction. These findings provide a basis for further research to elucidate the molecular evolutionary dynamics of individual population variability within the species and genus. The plastome reported here also provides an essential genomic reference for future work on population variation and species differentiation within *Zingiber*.

## Simple Summary

*Zingiber salarkhanii* is a plant found only in Bangladesh, and until now, its genetic information was largely unknown. In this study, we analyzed its complete chloroplast DNA to learn more about its identity and how it relates to other members of the ginger family. The circular genome measures 163,980 bp and contains 138 genes arranged in the typical quadripartite structure. Our findings show that it is closely linked to other *Zingiber* species and shares many traits common to this group. We also identified simple sequence repeats (SSRs) and variable hotspot regions that could be useful for future species identification and comparative studies. The plant is recognized for its attractive pink flowers and large fruit, and previous botanical studies have noted the presence of natural compounds of potential medicinal value. This work provides the first plastome resource for *Z. salarkhanii*, offering important information for taxonomic clarification, evolutionary assessment, and conservation planning. We also highlight the need to study additional *Zingiber* species to build a clearer understanding of their diversity and relationships within the ginger family.

## 1. Introduction

The *Zingiber* genus is found in a variety of environments, including the tropics, subtropics, and Far East Asia, as a member of the Zingiberaceae family [1]. The group comprises approximately 100 to 150 species, many of which are important for farming, medicine, and gardening [2]. Bangladesh is known for its rich biodiversity, with a vast array of plant species growing in its varied geographical regions [3]. Among these plant species, many are endemic to Bangladesh, meaning that they occur exclusively within the country [4]. In Bangladesh, eight species belonging to the genus *Zingiber* have been documented to date. These include *Z. capitatum* Roxb., *Z. montanum* (J. Koenig) Link ex A. Dietr., *Z. officinale* Roscoe, *Z. zerumbet* (L.) Roscoe ex Sm., *Z. roseum* Roxb., *Z. rubens* Roxb., *Z. salarkhanii* Rahman et Yusuf, and *Z. spectabile* Griff [5].

Several species of *Zingiber* have been identified based on both vegetative and floral traits and have been taxonomically classified. Nevertheless, certain distinguishing physical characteristics are often inconsistent and varied [6]. The vegetative elements of *Zingiber* species are visually similar during nonflowering seasons, complicating morphological differentiation across species at this stage [7]. Recently, many studies have employed molecular data to determine various *Zingiber* species.

**Figure 1:**
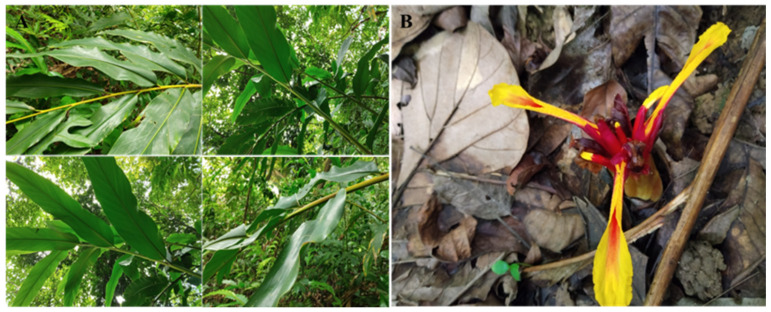
The Morphological picture of *Z. salarkhanii*. (**A**) A branch of a plant, (**B**) Flower.

*Z. salarkhanii* Rahman et Yusuf was first described from the hilly regions of Chittagong, Bangladesh, with the holotype collected from Sitakundu, Chandranath Hill (M. Yusuf & M.A. Rahman 825, BCSIRH). It has been described as having the following characteristics: the ligule is up to 0.5 cm in length and is 3-lobed; the spike is pinkish; the petals are lanceolate and uniformly pink; the labellum is 3-lobed, emarginated, and variegated; and the fruits are big, measuring 5.5–7.7 × 2.0–2.7 cm (Figure 1). It is considered Lower Risk (least concern) under IUCN (1994) due to its presence in multiple populations [8].

Furthermore, *Z. salarkhanii* is expected to exhibit the distinctive characteristics and pharmacological actions associated with the renowned Zingiberaceae family and is a promising source of phytochemicals that may alleviate pain and anxiety, inhibit cell growth, and act as antioxidants. Further research may reveal the scientific origins of its analgesic, anxiolytic, cytotoxic, and antioxidant effects [9], as well as its analgesic, anti-inflammatory, antibacterial, anticancer, and antidiabetic actions [10,11]. In recent years, *Zingiber* plants have been regarded as potential therapeutic agents for COVID-19 due to their antiviral properties [12,13]. It is valuable in aromatic rhizomes, which are used not only for cooking but also for conventional medicine and the development of diverse food and drink products.

Recently, molecular tools have played an increasingly important role in resolving the complex taxonomy of *Zingiber* [14]. Analyses based on nuclear *ITS* and the plastid *matK* region provided only weak resolution among several closely related species, including *Z. corallinum*, *Z. wrayi*, *Z. sulphureum*, *Z. gramineum*, *Z. ellipticum*, and an unidentified *Zingiber* sp. [15]. AFLP markers revealed that *Z. montanum* and *Z. zerumbet* are more closely related to each other than to *Z. officinale*, although the overall resolution of these analyses remains limited [7].

The informational resources on DNA barcoding detectors are essential for the efficacy of species identification [16,17]. The development of cp genomes, patterns and rates of nucleotide substitution, and phylogenetic linkages across terrestrial plant species have all been studied using cp genomes [18]. Unlike the mitochondrial and nuclear genomes, which are generally maternally inherited, the cp genome is largely non-recombinant and exhibits uniparental inheritance [19]. It offers a remarkable framework for examining species divergence and genome evolution. Most autotrophic land plants have conserved genes in terms of content, copy number, and structure. However, individual cp genes may experience different selective pressures as lineages adapt to various environments [20]. Genomic variability arising from evolutionary processes generates a multitude of molecular markers that are both useful and precise. These markers are useful for resolving phylogenetic relationships at many taxonomic levels [21].

In general land plants, the cp genome maintains a characteristic four-part structure, including a large single-copy (LSC) region, a small single-copy (SSC) region, and two inverted repeat (IR) segments. The genome typically spans 120–180 kb and encodes roughly 110–130 genes involved in photosynthesis, transcription, and other essential plastid functions [22]. However, very few cp genomes from *Zingiber* have been recorded in the Gene Bank, hindering molecular detection and the elucidation of evolutionary relationships among *Zingiber* species. The acquisition of cp genome sequences has become more efficient with the advent of high-throughput sequencing, offering a distinctive opportunity to investigate the phylogeny of *Zingiber* species and the evolution of the cp genome.

The genus *Zingiber* has made significant taxonomic contributions. However, due to a lack of complete cp genome sequences, proper species identification and precise phylogenetic interpretation remain elusive in the group. This gap is especially evident for *Z. salarkhanii*, an endemic Bangladeshi species with distinctive morphological traits but no available plastome data. Without genomic information, its evolutionary position, genetic distinctiveness, and affinities to related species remain unresolved. Given the proven value of cp genomes for addressing taxonomic uncertainty and reconstructing evolutionary histories in angiosperms, generating and characterizing the plastome of *Z. salarkhanii* is essential. In this study, we sequence, assemble, and analyze the complete cp genome of *Z. salarkhanii*, compare its structural and molecular features with other Zingiberaceae plastomes, and identify codon usage patterns, RNA editing sites, simple sequence repeats, long repeats, and mutational hotspots. We also infer its phylogenetic relationships within the genus. The findings offer valuable genomic resources for species identification, evolutionary assessment, and future phylogenetic studies in *Zingiber*.

## 2. Materials and Methods

### 2.1. Plant Materials, DNA Extraction

The following leaf sample was collected from Chattogram, Bangladesh (22°28′27.53″ N 91°47′07.56″ E) on 21 June 2023. The specimens were morphologically identified by Dr. Shaikh Bokhtear Uddin (email: bokhtear@cu.ac.bd), Professor, Department of Botany, University of Chittagong, Bangladesh, and a voucher sample was deposited in the King Abdulaziz University herbarium (Accession no: KAU-D6782). Genomic DNA was isolated from young leaf tissue using the DNeasy Plant Mini Kit (Qiagen, Hilden, Germany), and extraction was performed according to the manufacturer’s guidelines. DNA concentration and overall integrity were subsequently verified with a Qubit fluorometer and agarose gel electrophoresis.

### 2.2. Sequencing and Assembly

Library construction and sequencing were performed at BGI Genomics, Hong Kong, using base quality values on the DNBSEQ sequencing platform ranging from 2 to 43. The raw dataset was processed with SOAPnuke v2.1.7 [23] to remove adaptor contamination, low-quality sequences, and other impurities, resulting in 25 GB of high-quality 150 bp paired-end reads. Genome assembly was accomplished using Getorganelle 1.7.7.1, Spades 4.0 (k-mer threshold 21, 45, 65, 85, 105 and round 15) in Linux-6.11.0-8-generic-x86_64-with-glibc2.40 with Python 3.5.1 [24]. The entire cp genome sequence of *Z. teres* (NC_062457) was used as a guide to assemble the genome of *Z. salarkhanii*. For the species, a circular contig encompassing the entire cp genome was created.

### 2.3. Gene Annotation

Using GeSeq web-based tool, we annotated and predicted genes for whole cp genomes [25,26] and modified manually using Sequin 15.5 (accessed on 25 February 2024), where intron–exon boundaries, pseudogene predictions, and tRNA structures were checked for accuracy. The circular plot of the cp genome was generated using OGDRAW version 1.3.1. This was submitted to GenBank under accession number PV069723.

### 2.4. Codon Usage Bias and RNA Editing Site

MEGA12 was utilized to compute the GC content of the filtered coding sequences (CDSs). In contrast, the GC content at the first, second, and third positions of the codons (GC1, GC2, and GC3, respectively) were determined using the CUSP program in the EMBOSS explorer web-based tool. The RSCU (Relative synonymous codon usage) number denotes the ratio of the actual usage frequency of a codon to the anticipated usage frequency of all codons [27]. A codon with RSCU > 1 is considered preferentially used, whereas RSCU < 1 indicates reduced usage [28]. Additionally, the codon-usage indices T3s, C3s, A3s, and G3s, reflecting the frequencies of T, C, A, and G at the third positions of synonymous codons, were used to calculate RSCU. Codon-usage bias (CUB) was further assessed using the effective number of codons (ENC) and its corresponding ENC plot, which compares observed ENC values with GC3 content. ENC values range from 20 to 61, with lower values indicating stronger codon usage bias, while values approaching 61 indicate weak or no bias [29]. PR2-plot analysis was also applied to examine how mutation and natural selection shape CUB by evaluating nucleotide composition at the third codon position. Under the PR2 expectation, bases at this position should occur in equal proportions (A = T and C = G) [30]. If the distribution is not equal, it suggests unequal mutational pressure or selective constraints [31]. A neutrality plot (GC12–GC3) was used to evaluate and outline the codon usage patterns across the three codon positions. GC12 represents the average of GC1 and GC2 [32]. A regression figure with a slope of 0 signifies the absence of directional mutation pressure (indicating perfect selection limitations), while a slope of 1 indicates an identical mutation module between GC12 and GC3. To identify potential RNA editing sites in the ten cp genomes, protein-coding genes were analyzed using the online Predictive RNA editor for Plants (PREP) tool with a cutoff threshold of 0.8 [33]. In cp genome studies, the threshold is often used because it is deemed a good compromise between sensitivity and specificity [34]. Therefore, the threshold minimizes false positives while retaining true editing predictions. The upper and lower 5% of genes were designated as high- and low-expression datasets, respectively, and RSCU values for each dataset were computed using CodonW 1.4.2 [35]. Optimal codons were identified using the ΔRSCU approach, in which a codon is considered optimal when its ΔRSCU value is ≥0.08, and its RSCU in either the high- or low-expression dataset exceeds 1 [36].

### 2.5. SSR and Long Repeat Analyses

The local MISA algorithm was used to forecast the SSR [37], with the maximum allowable counts for mononucleotide, dinucleotide, trinucleotide, tetranucleotide, pentanucleotide, and hexanucleotide repetitions established at 10, 6, 4, 3, 3, and 3, respectively. Additionally, we employed REPuter web-based tool to analyze repeat sequences, encompassing complementary, forward, reverse, tandem, and palindromic repetitions. The minimum required repeat size was set as 30 bp with a repeat identity of 90% and a Hamming distance of 3 [38].

### 2.6. IR Contraction/Expansion and Genome Divergence

We analyzed the cp genome boundaries of *Z. salarkhanii* in comparison to those of nine other *Zingiber* species for LSC, SSC, and IRs, using matching annotations from CPJSdraw (version 1.0; Perl-based) [39]. The nine cp genomes were aligned to the *Z. salarkhanii* genome as the reference using the Shuffle-LAGAN mode of mVISTA web-based tool and a RankVISTA probability threshold of 0.5 (0 < *p* < 1). The software results necessitated manual adjustments to address overlapping gene names. The nucleotide diversity (Pi) of the cp genome was assessed in DnaSP v5.1 using a sliding-window approach, with a window length of 800 bp and a step size of 100 bp [40,41].

### 2.7. Phylogenetic Analysis in Cp Genomes

Cp genome sequences of 37 species, including *Z. salarkhanii*, were used for comparison using the MAFFT-7.526 software [42]. Then, a phylogenetic tree was created based on the maximum likelihood (ML) methods in MEGA 12 software [43], where the self-expansion value of each parameter was set to 1000.

## 3. Results

### 3.1. General Characteristics of Z. salarkhanii

**Figure 2:**
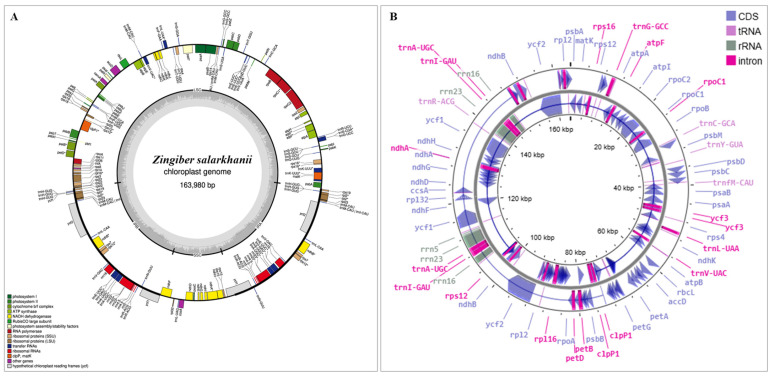
(**A**) Circular map of the *Zingiber salarkhanii* chloroplast genome. Genes oriented on the inner circle are transcribed anticlockwise, and those located on the outer circle are transcribed in the opposite (counterclockwise) direction. The dark grey inner ring represents GC content, while the lighter outer ring shows the corresponding AT content distribution throughout the genome. (**B**) Circular Genome View. The cp genome highlights coding regions (CDS), tRNAs, rRNAs, and intron-containing genes in a multi-ring display. The inner rings show variation in GC content and gene density across the genome, providing a comprehensive view of nucleotide composition and structural features.

The cp genome of *Z. salarkhanii* spans 163,980 base pairs and exhibits the typical quadripartite layout characteristic of maximum angiosperms (Figure 2). It comprises a large single-copy (LSC) region of 88,462 bp, a small single-copy (SSC) region of 15,890 bp, and two inverted repeat (IR) regions (IRa and IRb), each measuring 29,814 bp. The GC content is 36.91%, suggesting high conservation across *Zingiber* species in the cp genome. When observed at individual codon positions, the GC content showed a transparent gradient (GC1 > GC2 > GC3): 44.51%, 37.40%, and 28.82%, respectively. The observed pattern reflects codon usage bias and selective constraints within cp protein-coding genes. The cp genome of *Z. salarkhanii* comprises 138 annotated genes, including 90 protein-coding sequences (Unique 79 genes), 40 tRNA genes, 30 unique genes, eight rRNA genes, and four unique genes. Among these, 113 are unique, while 48 are duplicated within the inverted repeat (IR) regions, a genomic feature consistent with other members of the Zingiberaceae family (Table S1).

### 3.2. Codon Usage Bias and RNA Editing

The GC content at the first, second, and third codon positions (GC1, GC2, and GC3) remained below 50% in all cases, indicating that the cp genome is predominantly AT-rich. Codons that end in A or U are markedly preferred, and is a characteristic exhibited by most angiosperm plastomes. The GC content decreased distinctly from GC1 to GC3, indicating that selective pressures on codon usage differ across codon positions.

#### 3.2.1. RCSU and RFSC Analysis

Relative synonymous codon usage (RSCU) values were calculated to evaluate variations in codon usage among the genes. An RSCU value of 1 signifies a neutral codon use, when all synonymous codons for a given amino acid are employed with equal frequency. If it is greater than 1, it indicates preferential usage. If it is <1, it is underused. In *Z. salarkhanii*, it was observed that 30 codons had an RSCU value of more than one, as they were the most used codons. Furthermore, 90% of codons ended in A or U, indicating the AT-rich nature of the genome. In contrast, 34 codons displayed RSCU values below 1, of which 85.3% ended in G or C. Only one codon was highly overrepresented (RSCU = 2.00), and two codons were neutral (RSCU = 1.00). Of the amino acids, arginine (Arg) had the highest codon bias (an AGA value of 2.00, the most overrepresented codon) and the lowest frequency (a CGC value of 0.45). The findings demonstrated that methionine (Met) and tryptophan (Trp) did not exhibit bias (RSCU = 1.00), as each is recognized by a single codon. Serine had the highest diversity of synonymous codons, while tryptophan had the lowest (Table 1). The relative frequency of synonymous codons (RFSC) was calculated to determine the RSCU. RFSC offers the ratio of each synonymous codon to all codons that encode the same amino acid, offering additional insight into codon usage preference. The codons marked with asterisks (Table 1) are putative optimal codons defined by a high RSCU value and consistently high RFSC values in all genes.

#### 3.2.2. Analysis of Codon Usage Bias and Optimal Codon Patterns

**Figure 3:**
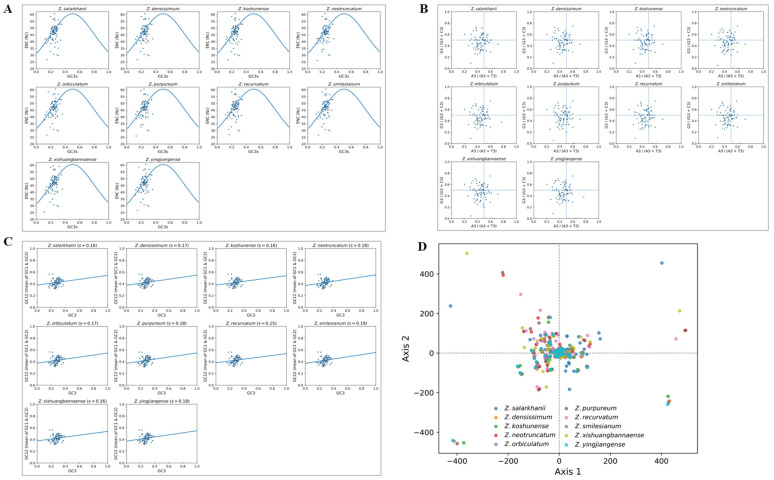
Codon usage patterns of *Z. salarkhanii* and the nine other species of cp genome; (**A**) ENc-plot, (**B**) PR2-plot, (**C**) Neutrality plot, (**D**) COA plot.

The ENC–GC3s graph strongly implies that natural selection is the primary factor influencing the codon usage patterns of ten *Zingiber* species. Most plastid genes are situated below the anticipated ENC–GC3s curve, indicating that strong selection outweighs the influence of neutral mutations. Most genes are not on or above the theoretical curve. This suggests that adaptive pressures, especially those associated with translational efficiency and gene expression optimization, play a dominant role in evolution. The ranges of GC3 and ENC values for the species are 0.2 to 0.5 and 30 to 55, respectively. This means that they have a moderate value of codon bias. The GC3 values of *Z. salarkhanlii*, which are about 0.2–0.45, and the corresponding ENC values, around 30–55, are comparable in pattern to those of other *Zingiber* species (Figure 3A). Together, these findings indicate that the evolution of *Zingiber* cp genomes has been predominantly driven by translational selection rather than mutational drift, a trend observed more generally in angiosperms.

The PR2 (Parity Rule 2) bias plots for ten *Zingiber* species showed significantly asymmetric nucleotide composition at the third codon position of plastid genes (Figure 3B). If there were complete parity, we would expect gene loci to be distributed around the coordinate (0.5, 0.5) for equal proportions of A versus T and G versus C. However, that is not the case. Among all species, gene point dispersal is away from the center. The wide dispersion of gene points indicates heterogeneous selective constraints among cp genes, suggesting that codon usage is shaped not only by mutational pressure but also by gene-specific selective forces related to functional conservation and translation efficiency. Most loci showed an overrepresentation of either purines (A, G) or pyrimidines (T, C). This systematic bias strongly signifies that codon usage in these genomes is influenced by both random mutation processes and directional selective pressures. The biases might be due to translation or strand-level mutation mechanisms. All panels showed a similar pattern, indicating that the codon bias pattern in *Zingiber* is conserved and non-random. This study confirms the occurrence of codon bias in *Zingiber*. It further our understanding of codon bias evolution and functionality in the angiosperm cp genome.

Neutrality plot analysis, comparing the GC content at the third codon position (GC3) with the mean GC content of the first and second positions (GC12), demonstrated that selection pressure played a dominant role in shaping codon usage patterns across all ten *Zingiber* species (Figure 3C). In *Z. salarkhanlii*, the slope of the regression on mutational bias, s = 0.16, indicated a low mutational bias for GC3 variation. Other species showed similar low slope values of 0.15–0.19, suggesting uniform evolution across the entire genus. The slopes of these were relatively low (s < 0.20), indicating that codon composition is determined by selection rather than mutational drift. The data points bunched tightly within a narrow GC3–GC12 range furnish further evidence for the existence of strong, conserved selection pressure acting on plastid genes and on the functional and translational optimization of *Zingiber* cp genomes.

The combined correspondence analysis (COA) of plastid genes from nine related species and *Z. salarkhanii* showed that all analyzed species were tightly clustered and overlapped extensively along the two major axes (Figure 3D). The dense clustering indicated high conservation and homogeneity in codon usage bias within this genus, a result arising from the genes of different lineages. Furthermore, it shows that sometimes selection pressure acts on similar codons, whereas the use of codons in recent lineages has been driven by mutation. Some genes are outliers with localized deviations in codon preference, which might be functionally important or imply an adaptive regulatory process. However, *Zingiber* plastid genomes show a high conservation of translational optimization strategies. These results reveal that purifying selection and functional constraint play a crucial role in maintaining the coherence of codon usage patterns. In addition, these results reveal evolutionary constraints that preserve the efficient functioning of plastid genomes in all members of the genus.

**Figure 4:**
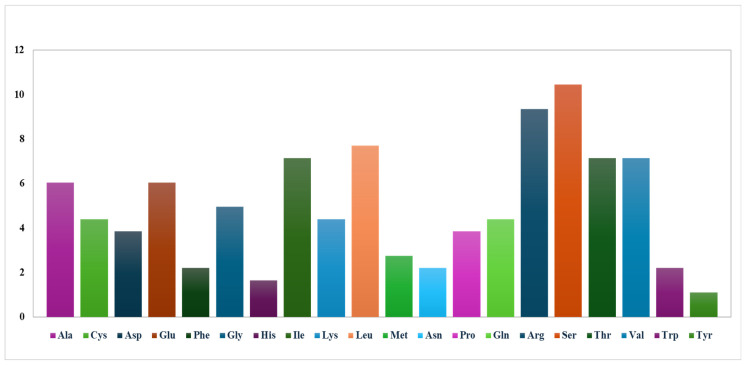
RSCU profiles of the 20 amino acids and stop codons based on all protein-coding sequences in the *Z. salarkhanii* cp genome.

A two-way chi-squared (χ^2^) contingency test was conducted to evaluate variations in codon usage among the genes, providing a statistical foundation for identifying optimal codons that could enhance the expression of essential proteins. Based on the RSCU analysis of *Z. salarkhanii*, 14 codons with RSCU values ranging from 1.3 to 1.6 were designated as high-frequency codons. Furthermore, comparative evaluation identified eighteen optimal codons (defined by ΔRSCU > 0.08 and RSCU > 1) across the genome (Table S2). These findings establish a set of preferred codons that may serve as a benchmark for codon optimization in future genetic and expression studies (Figure 4).

#### 3.2.3. RNA Editing Sites

**Figure 5:**
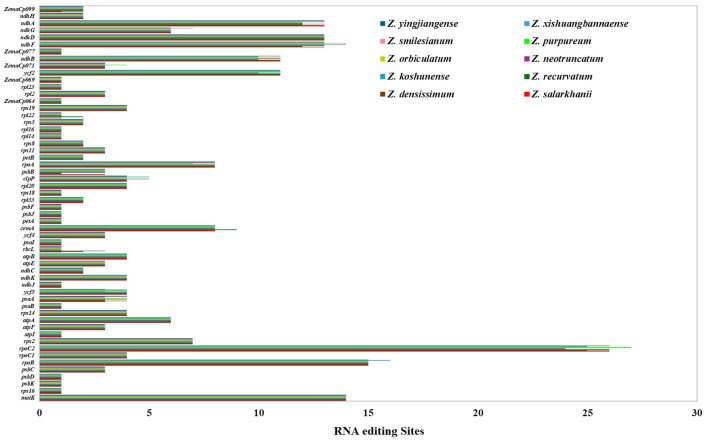
The predicted RNA editing site of the cp genome in *Z. salarkhanii* and nine other species.

In the cp genome of *Z. salarkhanii*, a total of 55 RNA editing sites involving C–to–U conversions were identified (Table S3). These sites were found on 49 protein-coding genes, suggesting RNA editing is pervasive in this genome. Based on the comparative dataset across the ten *Zingiber* species, *rpoC2* contained the highest number of editing sites (26) in *Z. salarkhanii*, a pattern consistent with *Z. recurvatum*, *Z. koshunense*, *Z. orbiculatum*, *Z. purpureum*, and *Z. smilesianum*, all of which showed 26–27 edits in this gene. The next most edited genes in *Z. salarkhanii* were *ndhB* (11–17 edits across species) and *matK* (14 edits in all species), followed by *ndhA*, *ndhD*, and *ndhF*, each with 13 editing sites, values also shared with most congeners. Similarly, *ycf2* exhibited 11 editing sites, matching nearly all species except *Z. neotruncatum*, which had 10. Most remaining genes displayed fewer than ten edits, and only minor species-specific differences were observed (e.g., *rbcL* with 1–3 edits, *petB* with 0–2 edits, *rpl22* with 0–2 edits). The amino acid substitution outcomes revealed that the two most common substitutions were serine-to-leucine and phenylalanine-to-leucine (Figure 5). The cp RNA editing is conserved in higher plants. All identified editing events in *Z. salarkhanii* were C-to-T transitions, occurring exclusively at the first and second codon positions, a pattern shared across the other nine *Zingiber* species. Overall, the editing profile of *Z. salarkhanii* closely aligns with that of *Z. densissimum*, *Z. recurvatum*, *Z. koshunense*, *Z. neotruncatum*, *Z. orbiculatum*, *Z. purpureum*, and *Z. smilesianum*, reflecting a highly conserved cp RNA editing system within the genus.

### 3.3. SSRs and Long Repeats Analyses

**Figure 6:**
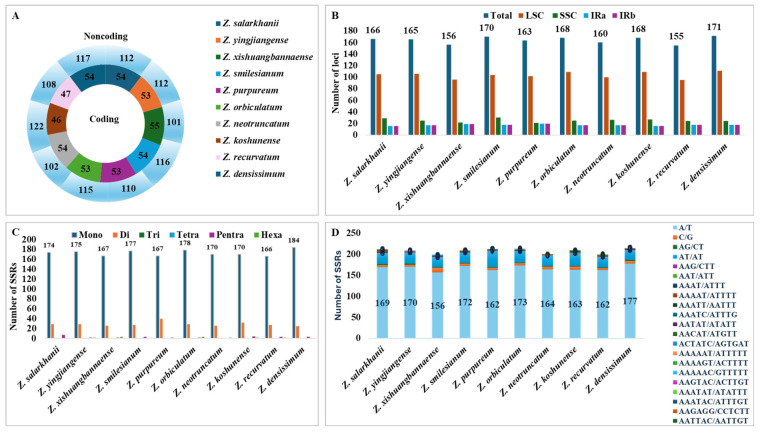
Comparison of simple sequence repeats (SSRs) across ten *Zingiber* chloroplast genomes, including *Z. salarkhanii*. (**A**) Distribution of SSRs between coding and non-coding regions. (**B**) Number of SSRs located in the LSC, SSC, and IR regions. (**C**) Variation in SSR motif types was identified among the species. (**D**) Frequency of SSRs across different repeat classes.

Simple Sequence Repeats (SSRs), often referred to as microsatellites, are short stretches of DNA made up of small nucleotide motifs, usually only 1 to 6 base pairs long, that repeat consecutively in tandem. The widespread presence of these components in the cp genome has facilitated species identification, marker development, and a wide range of phylogenetic and population genetic analyses. In the cp genome of *Z. salarkhanii*, most SSRs were in the LSC and SSC regions. Furthermore, these regions are among the most variable and functionally significant in the cp genome. According to the findings, the inverted repeat regions have comparatively fewer SSRs. The number of SSRs in coding loci ranged from 46 to 54 across ten *Zingiber* species. In comparison, there were 101–117 SSRs in noncoding loci. Specifically, *Z. salarkhanii* had 54 coding SSRs and 112 noncoding SSRs (Figure 6A,B). A total of 211 SSRs were detected in the genome. The cp genome contains 174 single-nucleotide, 29 double-nucleotide, 1 triple-nucleotide, and 7 pentanucleotide repeats, with no hexanucleotide repeats. Out of ten Zingiberaceae species selected for the current study, a range of total SSRs per genome was found to be 198 to 214 with prominent mononucleotide repeat (167 to 184 per species), followed by dinucleotides (25 to 40), tetranucleotides (0 to 1; detected in four species), trinucleotides (0 to 1; present in six species), pentanucleotides (1 to 7), and hexanucleotides (0 to 3; identified in seven species) (Figure 6C,D). Most mononucleotide SSRs consisted of A/T repeats and contributed between 68.42 and 75.44% of the total SSRs. In *Z. salarkhanii*, A/T repeats constituted 74.12%**,** followed by AT/AT dinucleotide repeats, which represented 10.09–16.67% of total SSRs across species. Other repeat types occurred at frequencies below 5%, indicating that A/T-rich SSRs are the dominant motif class in *Zingiberaceae* cp genomes.

Long repeats exceeding 30 bp are known to facilitate cp genome rearrangements and contribute to population-level genetic diversity, making them an important focus in cp genomics research. In this study, the repeated sequence patterns were analyzed across ten cp genomes of *Zingiberaceae* species.

**Figure 7:**
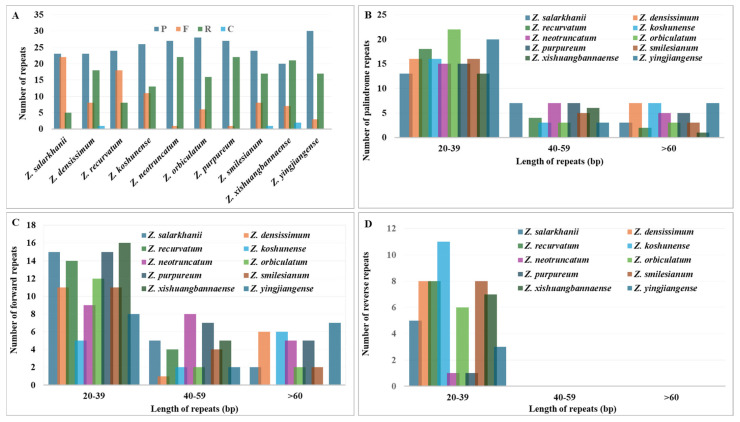
The occurrence and distribution of long repeat sequences within the cp genomes of ten *Zingiber* species. (**A**) Our survey revealed four main types of long repeats: palindromic (P), forward (F), reverse (R), and complementary (C). (**B**) We quantified the frequency with which palindromic repeats appeared across different length classes. (**C**) The same length-based assessment was performed for forward repeats to determine variation in their abundance. (**D**) We also evaluated the frequency of reverse repeats and how their occurrence changed with repeat length.

The cp genome of *Z. salarkhanii* contained a total of 50 long repeats, comprising 23 palindromic, 22 forward, and five reverse repeats, while no complement repeats were detected (Table S4). Across the ten *Zingiber* species examined, the abundance of the four repeat types varied. The number of palindromic repeats ranged from 20 to 30, reverse repeats from 5 to 22, and forward repeats from 3 to 22, whereas complement repeats were comparatively rare, ranging from 0 to 2 per species (Figure 7).

### 3.4. IR Contraction and Expansion Analyses

A detailed assessment of the LSC, IRs, and SSC boundary regions was performed across the ten *Zingiber* species. The inverted repeat regions (IRa and IRb) emerged as the most conserved sections of the cp genomes. Changes in the expansion or contraction of these IR boundaries are widely believed to influence differences in cp genome size among species. The size of the IR regions among the ten *Zingiber* cp genomes demonstrated minimal variation, ranging from 29,792 bp to 29,934 bp.

**Figure 8:**
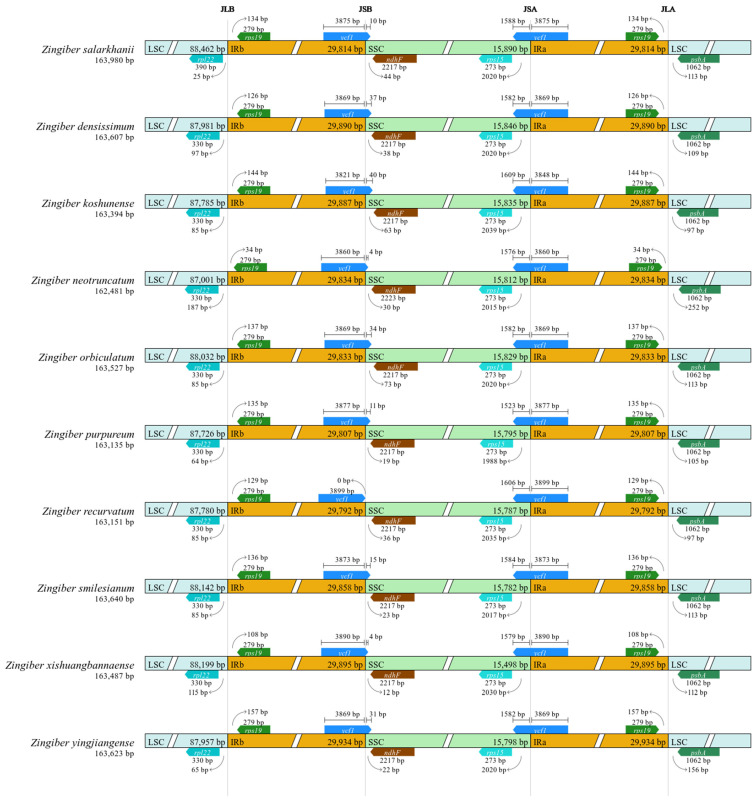
Illustrates the boundary relationships among the LSC, SSC, and IR regions in nine Zingiber chloroplast genomes, along with *Z. salarkhanii*. The schematic is not drawn to scale. Each colored block represents a whole gene or a gene fragment positioned near a junction.

In all *Zingiber* species, the *rpl22* and *rps19* genes were located near the LSC/IRb junctions, with distances between these genes and their respective boundaries ranging from 26 bp to 108 bp (Figure 8). Similarly, the *ycf1* and *ndhF* genes were located at the IRb/SSC boundaries in all ten species, with *ycf1* consistently at the end of the IRb region. Partial expansion of *ycf1* into the SSC region was observed across species, varying in length: approximately 3 kb in *Z. neotruncatum* and *Z. xishuangbannaense*; 10–16 kb in *Z. purpureum*, *Z. smilesianum*, and *Z. salarkhanii*; and 30–39 kb in *Z. recurvatum*, *Z. densissimum*, *Z. orbiculatum*, *Z. yingjiangense*, and *Z. koshunense*. The presence of this partial duplication results in the truncated pseudogene *ycf1*. The differences in *ycf1* length among species reflect slight IR expansion–contraction events that contribute to plastome size variation and shed light on the structural evolution of *Zingiber* plastomes.

The *ycf1* gene within the IRa region also showed size variation among the species, ranging from 3822 bp to 3891 bp. Additionally, the *rps19* and *psbA* genes were located near the IRa/LSC junction in all species. The distance between *rps19* and the IRa/LSC border ranged from 35 bp to 158 bp, whereas the *psbA* gene was separated from this boundary by approximately 97–113 bp in all ten *Zingiber* species (Figure 8). The *Zingiber* IR regions appear stable overall, but subtle shifts in their boundaries indicate that they are still evolving.

### 3.5. Genomic Comparative and Nucleotide Diversity Analyses

**Figure 9:**
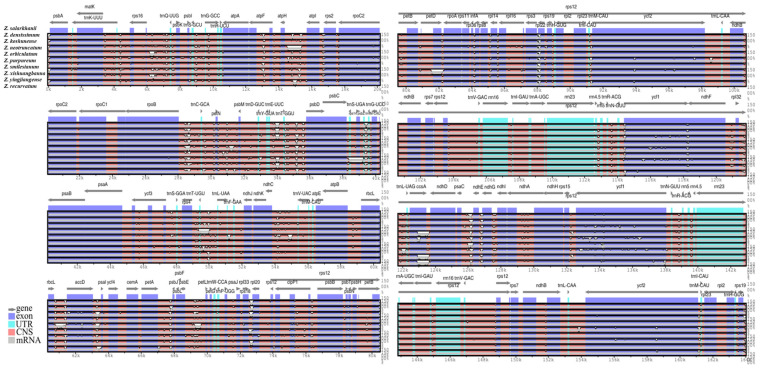
To represent an mVISTA comparison of the *Z. salarkhanii* cp genome with nine other *Zingiber* genomes. Grey arrows show the direction of gene transcription, while exons, UTRs, and conserved noncoding regions are displayed in dark blue, light blue, and pink, respectively. The vertical axis indicates the percentage of sequence identity, with the 100% identity line marking regions of complete conservation. White peaks highlight the sections where the genomes show the greatest sequence divergence.

A comparative assessment of the ten *Zingiber* cp genomes was performed using the mVISTA platform, with *Z. salarkhanii* selected as the reference genome. The assessment revealed that the LSC and SSC areas exhibited noticeably higher levels of variation than the more conserved IR regions. Additionally, the noncoding regions showed more nucleotide distinction than the coding regions, suggesting that intergenic spacers are more evolutionarily flexible. The regions that underwent the highest divergence within the coding regions were *psbA*, *rps16–psbK*, *atpF*, *atpH–atpI*, *rbcL–accD*, *psaI*, *petA–psbM*, *rps18–rpl20*, *rpl33–rpl20*, *petD–rpoA*, *rpl22*, *rrn5*, *ycf1*, *rpl32*, *psaC–ndhG*, and *rpl23*. On the other hand, the most variable noncoding regions included *trnQ–UUG*, *trnS–GCU–trnR–UCU*, *trnT–UGU–trnL–UAA*, *trnV–UAC*, *trnM–CAU*, *trnT–CAU*, *trnL–CAU*, *trnN–UAG*, *trnR–ACG*, *trnH–GUG*, and *trnM–CAU* (Figure 9).

**Figure 10:**
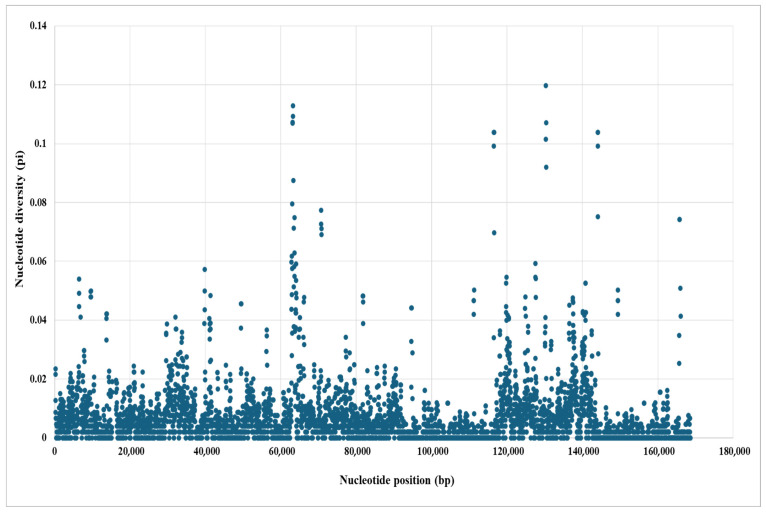
Sliding window analyses of ten *Zingiber* with *Z. salarkhanii* cp genomes. The nucleotide diversity (Pi) value of each window is shown on the *Y*-axis, and positions are shown on the *X*-axis.

The nucleotide diversity (π) analysis showed regions of sequence variation among the *Zingiber* cp genomes that are useful for finding molecular markers. To examine the distribution of nucleotide variation across the aligned genomes, a sliding-window analysis was performed. The overall π values ranged from 0 to 0.11289, indicating low genetic divergence, with few regions exhibiting higher variability. Eleven regions with π > 0.1 were identified as highly divergent, indicating hotspots of nucleotide variation within populations (Table S5). Nucleotide variability of *psaI*, *rps12*, and *ndhH* was found to be the highest. The gene *psaI* showed the highest level of nucleotide variability. All these hotspots are present in intergenic regions; thus, they are good candidates for developing DNA barcodes and other markers in phylogenetic and population-level studies in Zingiberaceae. The highly variable loci thus identified could serve as molecular markers and informative regions for phylogenetic and population genetic studies in Zingiberaceae (Figure 10).

### 3.6. Phylogenetic Analysis

To clarify the phylogenetic position of *Z. salarkhanii* within the Zingiberaceae family, a maximum-likelihood (ML) phylogenetic analysis was performed using the complete cp genome sequences of 37 Zingiberaceae species. Three additional species from the families Musaceae, Strelitziaceae, and Cannaceae, all belonging to the order Zingiberales, were included as outgroups to root the phylogenetic tree.

The resulting ML tree robustly resolved the Zingiberaceae family into two well-supported subfamilies, Zingiberoideae and Alpinioideae, each forming distinct clades (bootstrap support = 100%). Within Zingiberoideae, the tribe Zingibereae included five genera: *Curcuma*, *Zingiber*, *Boesenbergia*, *Roscoea*, and *Pommereschea*, all forming a monophyletic group with strong statistical support. The tribe Alpinieae, comprising *Amomum*, *Alpinia*, *Lanxangia*, and *Riedelia*, was similarly well supported (BS = 100%).

**Figure 11:**
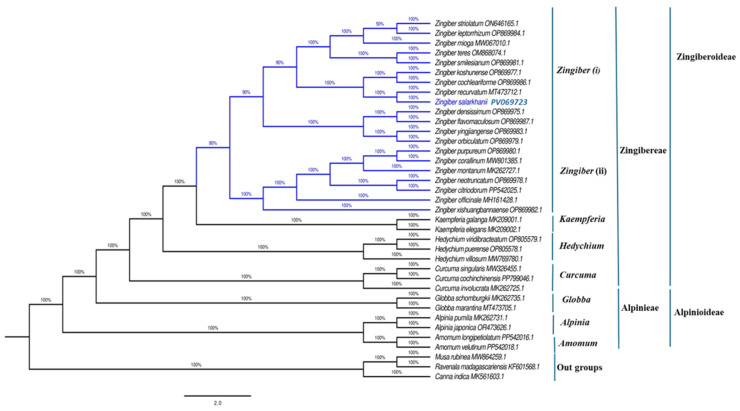
The maximum likelihood (ML) phylogenetic tree was created from the cp genome sequences of 37 species. The numerical values displayed beside each branch indicate the bootstrap support scores.

Within the genus *Zingiber*, *Z. salarkhanii* formed a strongly supported clade and was recovered as sister to *Z. recurvatum* (BS = 100%). This pair clustered closely with *Z. densissimum*, *Z. flavomaculosum*, *Z. yingjiangense*, *Z. orbiculatum*, and *Z. purpureum*, indicating a well-defined subgroup within the genus. This placement clearly establishes the evolutionary position of *Z. salarkhanii* and suggests it shares a recent common ancestor with *Z. recurvatum* (Figure 11).

## 4. Discussion

In this study, cp genomes of *Z. salarkhanii* were reported for the first time. Their genome size was 163,980 bp, the GC content was 36.91%, and the genome had a quadripartite structure. All the protein-coding genes, tRNA, and rRNA displayed high resemblance, consistent with other Zingiberoideae cp genomes [44]. The conservation of plastomes has been shown in many angiosperms, including Malvaceae and Araceae, where identical gene content and gene order have been found [45]. The GC content of *Z. salarkhanii* was GC1 (44.51%) > GC2 (37.40%) > GC3 (28.82%). The overall GC content of the genes of *Zingiber* followed the trend GC1 > GC2 > GC3, further implying a bias toward A and T at the three codon positions [35]. Cp genes that prefer selected codons for translational efficiency, particularly in those involved in photosynthesis, are believed to be subject to natural selection [46]. CUB reveals where species or genes originated, how they changed over time, and how they evolved, revealing differences in the DNA of different living things [47]. The number of common codons was 30 (RSCU > 1), whereas 34 identical codons (RSCU < 1) and 2 codons (RSCU = 1) exposed no bias in this species. We found that there was a larger number of CUB (RSCU > 1) in the cp genomes of *Zingiber* species, particularly among certain synonymous codons [48]. The cp genome of higher plants is illustrated by a pervasive phenomenon of high CUB [49].

The ENC plot for most genes in *Z. salarkhanii* differed markedly from the normal curve. This finding indicates that codon bias is primarily influenced by natural selection and other variables. The PR2-plot analysis revealed that the third position of the codon in most genes shows a preference for T and C, indicating that selection pressure substantially affects codon usage patterns. This indicates that selection, rather than mutation alone, plays a dominant role in shaping CUB in *Zingiber*, likely because cp genes must maintain efficient and accurate translation under varying environmental conditions [50]. Moreover, the PR2 and ENC analyses suggest that CUB in this species’ cp genome is shaped not only by natural selection but also by additional forces, including mutational pressure [51]. The neutral theory proposes that most substitutions at the third codon position are neutral or nearly neutral. When codon usage is shaped primarily by selection, GC3 values remain tightly constrained and show little correlation with GC1 and GC2 [52]. The neutrality plot analysis findings demonstrate that the CUB in the cp genome of *Z*. *salarkhanii* is primarily shaped by natural selection and other mechanisms, with comparatively little effect from mutational pressure. These findings combined indicate that selection pressure significantly influences codon choices [53]. The RSCU of *Z. salarkhanii* shows 14 high-frequency codons and a comparative analysis of 18 optimal codons in the species.

Fifty-five RNA editing sites were forecast in this species, distributed across 49 protein-coding genes, with the *rpoc2* gene having the highest number (26). In addition, some genes had 10–20 editing sites (*ycf2*, *ndhA*, *ndhD*, *ndhF*, *ndhB*, *matK)*, all of which were C-to-T transitions at codon locations 1 or 2. Interestingly, most codon conversions from serine (S) to leucine (L) and most RNA editing sites modified hydrophobic amino acids, such as leucine, isoleucine, tryptophan, tyrosine, valine, methionine, and phenylalanine [54,55].

SSRs were extensively distributed across cp genomes and have been used extensively in population genetics and molecular phylogenetics research [56]. *Z. salarkhanii* has 211 SSRs, including single trinucleotides, seven pentanucleotides, 29 dinucleotides, and 174 mononucleotides. Each of the ten Zingiberaceae species has 198–214 SSRs. Mononucleotide repeats in non-coding areas were the most prevalent, contributing to AT richness. These findings align with most recorded angiosperms [57,58,59]. Due to their high mutation rates, SSRs provide powerful molecular markers for population-level analyses and are particularly valuable for evaluating genetic diversity and informing conservation strategies for endemic species such as *Z. salarkhanii* [60].

A/T repeats made up 68.42–75.44% of mononucleotide SSRs, whereas AT/AT repeats accounted for 10.09–16.67%, and the rest were below 5%. The *Z. salarkhanii* cp genomes contained 23 palindromic repeats: 22 forward and five reverse, with no complement repeats. In addition, *Zingiber* species had more palindromic (20–30) and reverse (5–22) repeats, and fewer forward (3–22) repeats, and fewer complement (0–2) repeats. The SSRs and long repeats revealed in this work may be beneficial for molecular analysis, including assessments of genetic diversity, phylogenetic relationships, species identification, and evolutionary studies [61].

The expansion and contraction around the margins of inverted repeat regions of cp genomes are prevalent in angiosperms, potentially resulting in size differences, gene duplications or losses, and the emergence of pseudogenes [62]. All ten cp genomes in this study showed significant conservation, with the IR regions being the most conserved. The cp genomes of *Z. salarkhanii* included the *rpl22* and *rps19* genes, which have been identified near the boundaries of the LSC/IRB areas in all *Zingiber* species. Nevertheless, the *ycf1-ndhF* genes were situated near the margins of the IRB/SSC areas in all *Zingiber* species. Thus, changes in the LSC/IR boundaries may be the principal determinants of the contraction and expansion of IR zones in these Zingiberoideae species [55].

Divergent areas among ten Zingiberoideae species are acceptable for species identification at the subfamily and genus levels, as indicated by mVISTA, CGView, nucleotide diversity, and ML trees. These areas of significant diversity may serve as DNA markers for identifying *Zingiber* species and for phylogenetic research [45]. Among them, the coding regions were observed in the regions of *psbA*, *rps16-psbk*, *atpF*, *atpH-atpl*, *rbcL-accD*, *psal*, *petA-psbM*, *rps18-rpl20*, *rpl33-rpl20*, *petD-rpoA*, *rpl22*, *rrn5*, *ycf1*, *rpl32*, *psaC-ndhG*, and *rpl23*. For the non-coding regions, strongly divergent regions, including *trnQ-UUG*, *trnS-GCU-trnR-UCU*, *trnT-UGU-trnL-UAA*, *trnV-UAC*, *trnM-CAU*, *trnT-CAU*, *trnL-CAU*, *trnN-UAG*, *trnR-ACG*, and *trnH-GUG*, were reported as suitable for species identification. The nucleotide diversity showed that the Pi value for the entire *Zingiber* cp genome ranged from 0 to 0.11289. There were 11 areas that showed substantial variation (Pi > 0.1). Among these divergence hotspots, the coding regions *psaI* and *rps12* showed consistently high nucleotide variation across all species, making them suitable candidates for cpDNA barcodes due to their high evolutionary rate [44]. Moreover, they are capable of effectively distinguishing species within Zingiberaceae.

Phylogenetic analysis using cp genomes may provide a robust framework for elucidating the genetic links across species [63]. To regulate the phylogenetic relationships of AV in Zingiberales, we used ML analyses of nucleic acid sequences from 37 complete cp genomes to build a phylogenetic tree. The result showed that *Z. salarkhanii* and even Zingiberaceae species may have complex phylogenies and confirmed their relationship within the same group. The wider clade reflected the well-established pattern in Zingiberales, with Musaceae, Strelitziaceae, and Cannaceae as successive outgroups, showing the split at greater depth within the order. Understanding their evolutionary history would provide us with more useful insights into how to adapt to the environment and increase plant productivity. However, this study has limitations. Only one individual of *Z. salarkhanii* was sequenced, preventing us from making conclusions on intraspecific variation or hybridization. Cp genomes capture only the plant’s maternal lineage, not its paternal lineage [64]. Therefore, nuclear genomic analyses are required to clarify the species boundaries and evolutionary processes. Future work should therefore include population-level sampling, sequencing of the nuclear genome, and functional validation of RNA editing and diverging hotspots to better understand the adaptation, genetic diversity, and evolutionary history of this endemic.

## 5. Conclusions

In conclusion, the first complete cp genome of *Z. salarkhanii* provided in this work offers a valuable genomic reference for this poorly known endemic species. Through comparative analysis, we highlighted several variable regions and repeat-rich loci with strong potential to serve as markers and future DNA barcodes. The phylogenetic results clearly placed *Z. salarkhanii* in the core *Zingiber*. This will help refine the taxonomic position and improve our understanding of evolutionary relationships within the family. As we have limited genomic resources for *Zingiber* in chloroplasts, sequencing additional species will be important for enhancing comparative frameworks, developing trustworthy species-level markers, and addressing outstanding taxonomic and phylogenetic questions in Zingiberaceae.

## Figures and Tables

**Table 1:** RSCU and RFSC values of the codons in the cp genomes of *Z. salarkhanii*.

Amino Acid	Codon	Count	RSCU	RSFC	Amino Acid	Codon	Count	RSCU	RSFC
Phe	UUU	2411	1.26	0.63	Tyr	UAU	1800	1.4	0.70
	UUC	1425	0.74	0.37		UAC	779	0.6	0.30
Leu	UUA	1242	1.38	0.23	Stop	UAA *	1350	1.32	0.63
	UUG	1161	1.29	0.22		UAG *	784	0.76	0.37
	CUU	1066	1.19	0.20	His	CAU	976	1.42	0.71
	CUC	640	0.71	0.12		CAC	403	0.58	0.29
	CUA	794	0.88	0.15	Gln	CAA	1077	1.42	0.71
	CUG	494	0.55	0.09		CAG	444	0.58	0.29
Iso	AUU	1937	1.19	0.40	Asn	AAU	2032	1.43	0.72
	AUC	1142	0.7	0.23		AAC	807	0.57	0.29
	AUA	1804	1.11	0.37	Lys	AAA	2308	1.35	0.68
Met	AUG	1016	1	1.00		AAG	1099	0.65	0.33
Val	GUU	815	1.34	0.34	Asp	GAU	1143	1.48	0.74
	GUC	408	0.67	0.17		GAC	406	0.52	0.26
	GUA	799	1.31	0.3275	Glu	GAA	1296	1.37	0.7
	GUG	414	0.68	0.17		GAG	594	0.63	0.3
Ser	UCU	1202	1.42	0.31556	Cys	UGU	772	1.21	0.6
	UCC	1010	1.19	0.26444		UGC	507	0.79	0.4
	UCA	950	1.12	0.24889	Stop	UGA *	945	0.92	1.0
	UCG	657	0.77	0.17111	Trp	UGG	730	1	1.0
Pro	CCU	631	1.08	0.26933	Arg	CGU	377	0.7	0.2
	CCC	573	0.98	0.24439		CGC	244	0.45	0.2
	CCA	739	1.26	0.31421		CGA	548	1.01	0.4
	CCG	403	0.69	0.17207		CGG	363	0.67	0.2
Thr	ACU	778	1.22	0.305	Ser	AGU	752	0.89	0.2
	ACC	625	0.98	0.245		AGC	520	0.61	0.1
	ACA	752	1.18	0.295	Arg	AGA	1087	2	0.4
	ACG	396	0.62	0.155		AGG	634	1.17	0.3
Ala	GCU	471	1.31	0.32668	Gly	GGU	536	0.99	0.2
	GCC	330	0.92	0.22943		GGC	321	0.59	0.1
	GCA	441	1.23	0.30673		GGA	801	1.48	0.4
	GCG	197	0.55	0.13716		GGG	502	0.93	0.2

* stop codon.

## Data Availability

The chloroplast genome sequence obtained in this work has been submitted to GenBank (accession number: PV069723). The data will become publicly accessible after the manuscript is published.

## Supplementary Materials

The following supporting information can be downloaded at: https://www.mdpi.com/article/10.3390/biology15010014/s1, Table S1: Gene composition of *Z. salarkhanii* chloroplast genome; Table S2: RSCU (Relative Synonymous Codon Usage) and ΔRSCU (Difference in Relative Synonymous Codon Usage) values of the codons in chloroplast genomes of *Zingiber salarkhanii*; Table S3: Predicted C-to-U RNA editing sites in chloroplast protein-coding genes of *Z. salarkhanii*; Table S4: Positions, types, and genomic locations of long repeats identified in the chloroplast genome of *Z. salarkhanii*; Table S5: Nucleotide diversity (π) hotspot regions and their corresponding genes in the chloroplast genome of *Z. salarkhanii*.
