# Supplementary material for: Complete Chloroplast Genome Sequence of the Endemic and Medicinal Plant Zingiber salarkhanii: Comparative Analysis and Phylogenetic Relationships
Source: Biology (Basel). 2025 Dec 20;15(1):14. doi: 10.3390/biology15010014 (PMC12784768; doi:10.3390/biology15010014)
Supplement: Supplementary file 1 [file biology-15-00014-s001.zip › Table S1.pdf]

Table S1: Gene composition of *Z. salarkhanii* chloroplast genome.

**Category for genes**	**Groups of genes**	**Name of genes**
Photosynthesis-related genes	Significant unit of Rubisco	*rbcL*
	Photosystem I	*psaA, psaB, psaC, psal, psaJ*
	Assembly/constancy of photosystem I	*ycf3, ycf4*
	Photosystem II	*psbA, psbB, psbC, psbD, psbE, psbF, psbH, psbI, psbJ, psbK, psbL, psbM, psbN, psbT, psbZ*
	ATP synthase	*atpA atpB, atpE,atpF, atpH, atpI*
	Cytochrome b/f complex	*petA, petB, petD, petG, petL, petN*
	Cytochrome c synthesis	*ccsA*
	NADPH dehydrogenase	*ndhA, ndhB(x2), ndhC, ndhD, ndhE, ndhF, ndhG, ndhH, ndhl, ndhJ, ndhK*
Transcription and translation-related genes	Transcription	*rpoA, rpoB, rpoC1, rpoC2*
	Ribosomal proteins	*rps2, rps3, rps4, rps7(x2), rps8, rps11, rps12(x2), rps14, rps15, rps16, rps18, rps19(x2), rpl2, rpl14, rpl16, rpl20, rpl22, rpl23, rpl32, rpl33, rpl36*
	Translation initiation factor	*infA*
RNA genes	Ribosomal RNA	*rrn4.5(x2), rrn5(x2), rrn16(x2), rrn23(x2)*
	Transfer RNA	*trnA-UGC(x2), trnC-GCA, trnD-GUC, trnE-UUC, trnF-GAA, trnfM-CAU, trnG-GCC(x2), trnG-UCC(x2), trnH-GUG(x2), trnI-CAU(x2), trnI-GAU(x2), trnK-UUU(x3), trnL-CAA(x2), trnL-UAA, trnL-UAG, trnM-CAU, trnN-GUU(x2), trnP-UGG, trnQ-UUG, trnR-ACG(x2), trnR-UCU(x2), trnS-GCU, trnS-GGA, trnS-UGA, trnT-GGU(x2), trnT-UGU, trnV-GAC(x2), trnV-UAC, trnW-CCA, trnY-GUA*
Other genes	RNA processing	*matK*
	Carbon metabolism	*cemA*
	Fatty acid synthesis	*accD*
	Proteolysis	*clpP1*
Genes of unknown function	Conserved reading frame	*ycf1(x2), ycf2(x2)*
