# Supplementary material for: Complete Chloroplast Genome Sequence of the Endemic and Medicinal Plant Zingiber salarkhanii: Comparative Analysis and Phylogenetic Relationships
Source: Biology (Basel). 2025 Dec 20;15(1):14. doi: 10.3390/biology15010014 (PMC12784768; doi:10.3390/biology15010014)
Supplement: Supplementary file 1 [file biology-15-00014-s001.zip › Table S2.pdf]

Table S2: RSCU (Relative Synonymous Codon Usage) and ΔRSCU (Difference in Relative Synonymous Codon Usage) values of the codons in chloroplast genomes of *Zingiber salarkhanii*

Amino Acid	Codon	Count	RSCU high	Averge	RSCU low	ΔRSCU	RSFC	Amino Acid	Codon	Count	RSCU high	Averge	RSCU low	ΔRSCU	RSFC
Phe	UUU(F)	2411	1.26	1918	1.25704	0.00296	0.63	Tyr	UAU(Y)	1800	1.4	1289.5	1.39589	0.0041	0.70
	UUC(F)	1425	0.74	1918	0.74296	-0.003	0.37		UAC(Y)	779	0.6	1289.5	0.60411	-0.0041	0.30
Leu	UUA(L)	1242	1.38	899.5	0.72424	0.65576	0.23	Stop	UAA(*)	1350	1.32	1067	1.26523	0.0548	0.63
	UUG(L)	1161	1.29	899.5	0.77476	0.51524	0.22		UAG(*)	784	0.76	1067	0.73477	0.0252	0.37
	CUU(L)	1066	1.19	899.5	0.84381	0.34619	0.20	His	CAU(H)	976	1.42	689.5	1.41552	0.0045	0.71
	CUC(L)	640	0.71	899.5	1.40547	-0.6955	0.12		CAC(H)	403	0.58	689.5	0.58448	-0.0045	0.29
	CUA(L)	794	0.88	899.5	1.13287	-0.2529	0.15	Gln	CAA(Q)	1077	1.42	760.5	1.41617	0.0038	0.71
	CUG(L)	494	0.55	899.5	1.82085	-1.2709	0.09		CAG(Q)	444	0.58	760.5	0.58383	-0.0038	0.29
Iso	AUU(I)	1937	1.19	1627.67	0.8403	0.3497	0.40	Asn	AAU(N)	2032	1.43	1419.5	1.43149	-0.0015	0.72
	AUC(I)	1142	0.7	1627.67	1.42528	-0.7253	0.23		AAC(N)	807	0.57	1419.5	0.56851	0.0015	0.29
	AUA(I)	1804	1.11	1627.67	0.90225	0.20775	0.37	Lys	AAA(K)	2308	1.35	1703.5	1.35486	-0.0049	0.68
Met	AUG(M)	1016	1	1016	1	0	1.00		AAG(K)	1099	0.65	1703.5	0.64514	0.0049	0.33
Val	GUU(V)	815	1.34	609	0.74724	0.59276	0.34	Asp	GAU(D)	1143	1.48	774.5	1.47579	0.0042	0.74
	GUC(V)	408	0.67	609	1.49265	-0.8226	0.17		GAC(D)	406	0.52	774.5	0.52421	-0.0042	0.26
	GUA(V)	799	1.31	609	0.7622	0.5478	0.3275	Glu	GAA(E)	1296	1.37	945	1.37143	-0.0014	0.7
	GUG(V)	414	0.68	609	1.47101	-0.791	0.17		GAG(E)	594	0.63	945	0.62857	0.0014	0.3
Ser	UCU(S)	1202	1.42	954.75	0.7943	0.6257	0.31556	Cys	UGU(C)	772	1.21	639.5	1.20719	0.0028	0.6
	UCC(S)	1010	1.19	954.75	0.9453	0.2447	0.26444		UGC(C)	507	0.79	639.5	0.79281	-0.0028	0.4
	UCA(S)	950	1.12	954.75	1.005	0.115	0.24889	Stop	UGA(*)	945	0.92	837.5	1.12836	-0.2084	1.0
	UCG(S)	657	0.77	954.75	1.4532	-0.6832	0.17111	Trp	UGG(W)	730	1	837.5	0.87164	0.1284	1.0
Pro	CCU(P)	631	1.08	586.5	0.92948	0.15052	0.26933	Arg	CGU(R)	377	0.7	310.5	1.21417	-0.5142	0.2
	CCC(P)	573	0.98	586.5	1.02356	-0.0436	0.24439		CGC(R)	244	0.45	310.5	0.78583	-0.3358	0.2

	CCA(P)	739	1.26	586.5	0.79364	0.46636	0.31421		CGA(R)	548	1.01	455.5	1.20307	-0.1931	0.4
	CCG(P)	403	0.69	586.5	1.45533	-0.7653	0.17207		CGG(R)	363	0.67	455.5	0.79693	-0.1269	0.2
Thr	ACU(T)	778	1.22	637.75	0.81973	0.40027	0.305	Ser	AGU(S)	752	0.89	636	1.18239	-0.2924	0.2
	ACC(T)	625	0.98	637.75	1.0204	-0.0404	0.245		AGC(S)	520	0.61	636	0.81761	-0.2076	0.1
	ACA(T)	752	1.18	637.75	0.84807	0.33193	0.295	Arg	AGA(R)	1087	2	860.5	1.26322	0.7368	0.4
	ACG(T)	396	0.62	637.75	1.61048	-0.9905	0.155		AGG(R)	634	1.17	634	1	0.1700	0.3
Ala	GCU(A)	471	1.31	359.75	0.7638	0.5462	0.32668	Gly	GGU(G)	536	0.99	540	0.99259	-0.0026	0.2
	GCC(A)	330	0.92	359.75	1.09015	-0.1702	0.22943		GGC(G)	321	0.59	540	0.59444	-0.0044	0.1
	GCA(A)	441	1.23	359.75	0.81576	0.41424	0.30673		GGA(G)	801	1.48	540	1.48333	-0.0033	0.4
	GCG(A)	197	0.55	359.75	1.82614	-1.2761	0.13716		GGG(G)	502	0.93	540	0.92963	0.0004	0.2
