# Supplementary material for: Complete Chloroplast Genome Sequence of the Endemic and Medicinal Plant Zingiber salarkhanii: Comparative Analysis and Phylogenetic Relationships
Source: Biology (Basel). 2025 Dec 20;15(1):14. doi: 10.3390/biology15010014 (PMC12784768; doi:10.3390/biology15010014)
Supplement: Supplementary file 1 [file biology-15-00014-s001.zip › Table S3.pdf]

Table S3. Predicted C-to-U RNA editing sites in chloroplast protein-coding genes of *Z. salarkhanii*.

Gene	Nucleotide position	Amino acid position	Triplet position within codon	Base conversion	Codon change	Amino acid conversion
matK	103	35	1	C→U	CAU→UAU	H→Y
	380	127	2	C→U	UCU→UUU	S→F
	556	186	1	C→U	CAC→UAC	H→Y
	580	194	1	C→U	CUC→UUC	L→F
	589	197	1	C→U	CCG→UCG	P→S
	689	230	2	C→U	CCU→CUU	P→L
	733	245	1	C→U	CUU→UUU	L→F
	772	258	1	C→U	CUU→UUU	L→F
	910	304	1	C→U	CAC→UAC	H→Y
	1037	346	2	C→U	UCG→UUG	S→L
	1112	371	2	C→U	CCG→CUG	P→L
	1250	417	2	C→U	UCU→UUU	S→F
	1355	452	2	C→U	GCA→GUA	A→V
	1480	494	1	C→U	CGU→UGU	R→C
rps16	143	48	2	C→U	UCA→UUA	S→L
psbK	34	12	1	C→U	CUU→UUU	L→F
psbD	626	209	2	C→U	GCU→GUU	A→V
psbC	8	3	2	C→U	ACC→AUC	T→I
	301	101	1	C→U	CCG→UCG	P→S
	854	285	2	C→U	ACC→AUC	T→I
rpoB	247	83	1	C→U	CCC→UCC	P→S
	467	156	2	C→U	UCA→UUA	S→L
	545	182	2	C→U	UCG→UUG	S→L
	560	187	2	C→U	UCG→UUG	S→L
	704	235	2	C→U	CCA→CUA	P→L
	899	300	2	C→U	ACA→AUA	T→I
	1028	343	2	C→U	GCA→GUA	A→V
	1097	366	2	C→U	ACA→AUA	T→I
	1126	376	1	C→U	CAU→UAU	H→Y
	1760	587	2	C→U	GCU→GUU	A→V
	1798	600	1	C→U	CCU→UCU	P→S
	2128	710	1	C→U	CCU→UCU	P→S
	2314	772	1	C→U	CUU→UUU	L→F
	2441	814	2	C→U	UCA→UUA	S→L
	3224	1075	2	C→U	GCU→GUU	A→V
rpoC1	526	176	1	C→U	CCA→UCA	P→S
	617	206	2	C→U	UCA→UUA	S→L

	1291	431	1	C→U	CGG→UGG	R→W
	182	61	2	C→U	UCU→UUU	S→F
rpoC2	20	7	2	C→U	CCG→CUG	P→L
	256	86	1	C→U	CAU→UAU	H→Y
	259	87	1	C→U	CAU→UAU	H→Y
	349	117	1	C→U	CCU→UCU	P→S
	385	129	1	C→U	CAU→UAU	H→Y
	1378	460	1	C→U	CAC→UAC	H→Y
	1516	506	1	C→U	CAU→UAU	H→Y
	1547	516	2	C→U	UCU→UUU	S→F
	1598	533	2	C→U	UCU→UUU	S→F
	1631	544	2	C→U	UCA→UUA	S→L
	1646	549	2	C→U	ACC→AUC	T→I
	1780	594	1	C→U	CCA→UCA	P→S
	1840	614	1	C→U	CUU→UUU	L→F
	2414	805	2	C→U	CCG→CUG	P→L
	2774	925	2	C→U	UCG→UUG	S→L
	2909	970	2	C→U	UCA→UUA	S→L
	3074	1025	2	C→U	UCG→UUG	S→L
	3151	1051	1	C→U	CCU→UCU	P→S
	3254	1085	2	C→U	UCA→UUA	S→L
	3281	1094	2	C→U	CCA→CUA	P→L
	3356	1119	2	C→U	CCU→CUU	P→L
	3403	1135	1	C→U	CAU→UAU	H→Y
	3529	1177	1	C→U	CCC→UUC	P→F
	3530		2	C→U		
	4142	1381	2	C→U	UCA→UUA	S→L
	4540	1514	1	C→U	CAU→UAU	H→Y
rps2	71	24	2	C→U	ACU→AUU	T→I
	134	45	2	C→U	ACA→AUA	T→I
	248	83	2	C→U	UCA→UUA	S→L
	301	101	1	C→U	CUU→UUU	L→F
	329	110	2	C→U	ACU→AUU	T→I
	388	130	1	C→U	CUC→UUC	L→F
	668	223	2	C→U	UCC→UUC	S→F
atpI	629	210	2	C→U	UCA→UUA	S→L
atpF	248	83	2	C→U	ACU→AUU	T→I
	388	130	1	C→U	CAU→UAU	H→Y
	92	31	2	C→U	CCA→CUA	P→L
atpA	17	6	2	C→U	GCC→GUC	A→V
	86	29	2	C→U	ACC→AUC	T→I
	263	88	2	C→U	UCC→UUC	S→F
	683	228	2	C→U	ACG→AUG	T→M

	863	288	2	C→U	CCA→CUA	P→L
	914	305	2	C→U	UCA→UUA	S→L
rps14	80	27	2	C→U	UCA→UUA	S→L
	158	53	2	C→U	CCA→CUA	P→L
	226	76	1	C→U	CAU→UAU	H→Y
	271	91	1	C→U	CAU→UAU	H→Y
psaB	655	219	1	C→U	CAU→UAU	H→Y
psaA	16	6	1	C→U	CCG→UCG	P→S
	1438	480	1	C→U	CUU→UUU	L→F
	1460	487	2	C→U	ACC→AUC	T→I
ycf3	185	62	2	C→U	ACG→AUG	T→M
	191	64	2	C→U	CCA→CUA	P→L
	407	136	2	C→U	UCC→UUC	S→F
	44	15	2	C→U	UCU→UUU	S→F
ndhJ	131	44	2	C→U	UCA→UUA	S→L
ndhK	617	206	2	C→U	UCA→UUA	S→L
	625	209	1	C→U	CCU→UCU	P→S
	664	222	1	C→U	CCC→UCC	P→S
	682	228	1	C→U	CAG→UAG	Q→*
ndhC	71	24	2	C→U	GCA→GUA	A→V
	323	108	2	C→U	UCA→UUA	S→L
atpE	185	62	2	C→U	GCU→GUU	A→V
	227	76	2	C→U	ACC→AUC	T→I
	374	125	2	C→U	ACA→AUA	T→I
atpB	257	86	2	C→U	ACG→AUG	T→M
	389	130	2	C→U	UCU→UUU	S→F
	403	135	1	C→U	CCC→UCC	P→S
	1184	395	2	C→U	UCA→UUA	S→L
rbcL	137	46	2	C→U	CCU→CUU	P→L
psaI	85	29	1	C→U	CAU→UAU	H→Y
ycf4	254	85	2	C→U	UCG→UUG	S→L
	452	151	2	C→U	GCC→GUC	A→V
	553	185	1	C→U	CUU→UUU	L→F
cemA	28	10	1	C→U	CUU→UUU	L→F
	32	11	2	C→U	CCC→CUC	P→L
	41	14	2	C→U	GCA→GUA	A→V
	44	15	2	C→U	UCU→UUU	S→F
	98	33	2	C→U	CCU→CUU	P→L
	275	92	2	C→U	ACA→AUA	T→I
	424	142	1	C→U	CUC→UUC	L→F
	577	193	1	C→U	CUU→UUU	L→F
petA	44	15	2	C→U	ACU→AUU	T→I
psbJ	59	20	2	C→U	CCU→CUU	P→L

psbF	77	26	2	C→U	UCU→UUU	S→F
rpl33	47	16	2	C→U	ACC→AUC	T→I
	56	19	2	C→U	GCC→GUC	A→V
rps18	95	32	2	C→U	UCC→UUC	S→F
rpl20	86	29	2	C→U	UCG→UUG	S→L
	131	44	2	C→U	GCU→GUU	A→V
	290	97	2	C→U	CCU→CUU	P→L
	308	103	2	C→U	UCA→UUA	S→L
clpP	209	70	2	C→U	CCC→CUC	P→L
	274	92	1	C→U	CAU→UAU	H→Y
	496	166	1	C→U	CUU→UUU	L→F
	559	187	1	C→U	CAC→UAC	H→Y
psbB	152	51	2	C→U	GCU→GUU	A→V
	475	159	1	C→U	CGG→UGG	R→W
	668	223	2	C→U	CCA→CUA	P→L
rpoA	200	67	2	C→U	UCU→UUU	S→F
	301	101	1	C→U	CAU→UAU	H→Y
	368	123	2	C→U	UCA→UUA	S→L
	482	161	2	C→U	CCA→CUA	P→L
	527	176	2	C→U	UCU→UUU	S→F
	545	182	2	C→U	GCG→GUG	A→V
	550	184	1	C→U	CAU→UAU	H→Y
	830	277	2	C→U	UCA→UUA	S→L
rps11	53	18	2	C→U	UCA→UUA	S→L
	179	60	2	C→U	UCU→UUU	S→F
	214	72	1	C→U	CCA→UCA	P→S
rps8	80	27	2	C→U	GCA→GUA	A→V
	223	75	1	C→U	CAU→UAU	H→Y
rpl14	208	70	1	C→U	CUC→UUC	L→F
rpl16	107	36	2	C→U	GCA→GUA	A→V
rps3	488	163	2	C→U	ACA→AUA	T→I
	601	201	1	C→U	CAU→UAU	H→Y
rpl22	95	32	2	C→U	UCG→UUG	S→L
rps19	44	15	2	C→U	UCG→UUG	S→L
	68	23	2	C→U	ACG→AUG	T→M
	221	74	2	C→U	GCA→GUA	A→V
	244	82	1	C→U	CAC→UAC	H→Y
	44	15	2	C→U	UCG→UUG	S→L
	68	23	2	C→U	ACG→AUG	T→M
	221	74	2	C→U	GCA→GUA	A→V
	244	82	1	C→U	CAC→UAC	H→Y
ZemaCp064	154	52	1	C→U	CAU→UAU	H→Y
rpl2	506	169	2	C→U	UCG→UUG	S→L

	2	1	2	C→U	ACG→AUG	T→M
	29	10	2	C→U	ACC→AUC	T→I
rpl23	71	24	2	C→U	UCU→UUU	S→F
ZemaCp069	103	35	1	C→U	CGA→UGA	R→*
ycf2	176	59	2	C→U	ACU→AUU	T→I
	307	103	1	C→U	CAC→UAC	H→Y
	380	127	2	C→U	GCA→GUA	A→V
	455	152	2	C→U	UCA→UUA	S→L
	548	183	2	C→U	GCA→GUA	A→V
	629	210	2	C→U	UCU→UUU	S→F
	724	242	1	C→U	CAA→UAA	Q→*
	23	8	2	C→U	UCC→UUC	S→F
	85	29	1	C→U	CCG→UCG	P→S
	136	46	1	C→U	CUC→UUC	L→F
	223	75	1	C→U	CAU→UAU	H→Y
ZemaCp071	299	100	2	C→U	UCC→UUC	S→F
	343	115	1	C→U	CCU→UUU	P→F
	344		2	C→U		
ndhB	149	50	2	C→U	UCA→UUA	S→L
	467	156	2	C→U	UCA→UUA	S→L
	542	181	2	C→U	ACG→AUG	T→M
	611	204	2	C→U	UCA→UUA	S→L
	704	235	2	C→U	UCC→UUC	S→F
	737	246	2	C→U	CCA→CUA	P→L
	149	50	2	C→U	UCA→UUA	S→L
	467	156	2	C→U	UCA→UUA	S→L
	542	181	2	C→U	ACG→AUG	T→M
	611	204	2	C→U	UCA→UUA	S→L
	704	235	2	C→U	UCC→UUC	S→F
	737	246	2	C→U	CCA→CUA	P→L
	830	277	2	C→U	UCA→UUA	S→L
	836	279	2	C→U	UCA→UUA	S→L
	1193	398	2	C→U	UCA→UUA	S→L
	1255	419	1	C→U	CAU→UAU	H→Y
	1481	494	2	C→U	CCA→CUA	P→L
ZemaCp077	193	65	1	C→U	CCU→UCU	P→S
ndhF	56	19	2	C→U	ACU→AUU	T→I
	62	21	2	C→U	UCA→UUA	S→L
	290	97	2	C→U	UCA→UUA	S→L
	377	126	2	C→U	GCU→GUU	A→V
	509	170	2	C→U	ACU→AUU	T→I
	671	224	2	C→U	UCA→UUA	S→L
	1369	457	1	C→U	CAU→UAU	H→Y

	1420	474	1	C→U	CAU→UAU	H→Y
	1450	484	1	C→U	CCA→UCA	P→S
	1558	520	1	C→U	CCA→UCA	P→S
	1592	531	2	C→U	UCU→UUU	S→F
	2024	675	2	C→U	ACC→AUC	T→I
	2189	730	2	C→U	GCA→GUA	A→V
ndhD	47	16	2	C→U	UCC→UUC	S→F
	130	44	1	C→U	CUC→UUC	L→F
	145	49	1	C→U	CAC→UAC	H→Y
	157	53	1	C→U	CAU→UAU	H→Y
	265	89	1	C→U	CCC→UCC	P→S
	383	128	2	C→U	CCA→CUA	P→L
	532	178	1	C→U	CUU→UUU	L→F
	878	293	2	C→U	UCG→UUG	S→L
	947	316	2	C→U	ACA→AUA	T→I
	1193	398	2	C→U	UCA→UUA	S→L
	1394	465	2	C→U	UCA→UUA	S→L
	1424	475	2	C→U	ACU→AUU	T→I
	1475	492	2	C→U	GCU→GUU	A→V
ndhG	20	7	2	C→U	ACA→AUA	T→I
	155	52	2	C→U	UCA→UUA	S→L
	158	53	2	C→U	UCG→UUG	S→L
	179	60	2	C→U	GCU→GUU	A→V
	302	101	2	C→U	UCA→UUA	S→L
	422	141	2	C→U	GCU→GUU	A→V
ndhA	563	188	2	C→U	UCA→UUA	S→L
	773	258	2	C→U	GCU→GUU	A→V
	862	288	1	C→U	CCU→UUU	P→F
	863		2	C→U		
	868	290	1	C→U	CUU→UUU	L→F
	919	307	1	C→U	CUU→UUU	L→F
	968	323	2	C→U	ACA→AUA	T→I
	1070	357	2	C→U	UCU→UUU	S→F
	50	17	2	C→U	UCG→UUG	S→L
	70	24	1	C→U	CAU→UAU	H→Y
	361	121	1	C→U	CUU→UUU	L→F
	392	131	2	C→U	UCU→UUU	S→F
	473	158	2	C→U	UCA→UUA	S→L
ndhH	10	4	1	C→U	CCG→UCG	P→S
	443	148	2	C→U	GCC→GUC	A→V
ZemaCp099	118	40	1	C→U	CAC→UAC	H→Y
	118	40	1	C→U	CAC→UAC	H→Y
