# Supplementary material for: Complete Chloroplast Genome Sequence of the Endemic and Medicinal Plant Zingiber salarkhanii: Comparative Analysis and Phylogenetic Relationships
Source: Biology (Basel). 2025 Dec 20;15(1):14. doi: 10.3390/biology15010014 (PMC12784768; doi:10.3390/biology15010014)
Supplement: Supplementary file 1 [file biology-15-00014-s001.zip › Table S4.pdf]

Table S4. Positions, types, and genomic locations of long repeats identified in the chloroplast genome of *Z. salarkhanii*.

29814	88462	P	29814	134166	0	0.00E+00
96	91168	F	96	91218	0	1.20E-48
96	91168	P	96	161128	0	1.20E-48
96	91218	P	96	161178	0	1.20E-48
96	161128	F	96	161178	0	1.20E-48
56	35183	P	56	35183	0	1.46E-24
52	10	P	52	10	0	3.73E-22
46	19992	P	46	19992	0	1.53E-18
46	91168	F	46	91268	0	1.53E-18
46	91168	P	46	161128	0	1.53E-18
46	91268	P	46	161228	0	1.53E-18
46	161128	F	46	161228	0	1.53E-18
43	126140	F	43	126161	0	9.77E-17
42	93868	F	42	93889	0	3.91E-16
42	93868	P	42	158511	0	3.91E-16
42	93889	P	42	158532	0	3.91E-16
42	158511	F	42	158532	0	3.91E-16
38	67034	P	38	67034	0	1.00E-13
33	82161	P	33	82196	0	1.02E-10
32	39390	F	32	39408	0	4.10E-10
28	15052	R	28	15052	0	1.05E-07
28	30411	R	28	30411	0	1.05E-07
28	67775	P	28	67827	0	1.05E-07
27	67728	F	27	67754	0	4.20E-07
26	14901	R	26	14901	0	1.68E-06
26	28429	F	26	28455	0	1.68E-06
26	126817	F	26	126847	0	1.68E-06
25	61356	F	25	61381	0	6.72E-06
25	123521	P	25	123550	0	6.72E-06
24	7149	F	24	7172	0	2.69E-05
24	15151	P	24	15151	0	2.69E-05
24	28641	F	24	28665	0	2.69E-05
24	32936	F	24	32951	0	2.69E-05
24	85114	F	24	85138	0	2.69E-05
24	126206	P	24	126206	0	2.69E-05
23	14929	R	23	14929	0	1.07E-04
23	39407	P	23	39438	0	1.07E-04
22	8718	P	22	48052	0	4.30E-04
22	8974	F	22	8995	0	4.30E-04
22	28619	F	22	28663	0	4.30E-04
22	39390	P	22	39438	0	4.30E-04
22	119721	P	22	119721	0	4.30E-04
22	121405	P	22	121405	0	4.30E-04

22	121700	P	22	121700	0	4.30E-04
22	122967	P	22	122967	0	4.30E-04
22	126140	F	22	126182	0	4.30E-04
21	8355	R	21	8355	0	1.72E-03
21	13082	F	21	13102	0	1.72E-03
21	28549	F	21	28688	0	1.72E-03
21	34493	F	21	34514	0	1.72E-03

Palindrome

20-40	13
40-60	7
>60	3
Total	23

Forward

20-40	15
40-60	5
>60	2
Total	22

Reverse

20-40	5
40-60	0
>60	0
Total	5
