# Supplementary material for: Complete Chloroplast Genome Sequence of the Endemic and Medicinal Plant Zingiber salarkhanii: Comparative Analysis and Phylogenetic Relationships
Source: Biology (Basel). 2025 Dec 20;15(1):14. doi: 10.3390/biology15010014 (PMC12784768; doi:10.3390/biology15010014)
Supplement: Supplementary file 1 [file biology-15-00014-s001.zip › Table S5.pdf]

Table S5. Nucleotide diversity (π) hotspot regions and their corresponding genes in the chloroplast genome of *Z. salarkhanii*.

0	600	107	1169	*psbA*
6800	7400	7250	7322	*trnQ-UUG*
9600	10200	9697	10472	*trnG-GCC*
9800	10400	9697	10472	*trnG-GCC*
15800	16400	15683	16427	*atpI*
16000	16600	15683	16427	*atpI*
16200	16800	16709	17420	*rps2*
16200	16800	15683	16427	*atpI*
16400	17000	16709	17420	*rps2*
16400	17000	15683	16427	*atpI*
16600	17200	16709	17420	*rps2*
32200	32800	32714	32788	*trnD-GUC*
32400	33000	32714	32788	*trnD-GUC*
32600	33200	32714	32788	*trnD-GUC*
36600	37200	35690	36752	*psbD*
36600	37200	36699	38121	*psbC*
36800	37400	36699	38121	*psbC*
37000	37600	36699	38121	*psbC*
56400	57000	56478	56886	*atpE*
56400	57000	56882	58370	*atpB*
56600	57200	56478	56886	*atpE*
56600	57200	56882	58370	*atpB*
63200	63800	63502	63613	*psaI*
63400	64000	63502	63613	*psaI*
63400	64000	63958	64513	*ycf4*
63600	64200	63502	63613	*psaI*
63600	64200	63958	64513	*ycf4*
63800	64400	63958	64513	*ycf4*
64000	64600	63958	64513	*ycf4*
64200	64800	63958	64513	*ycf4*
95200	95800	91422	98286	*ycf2*
95200	95800	73892	103733	*rps12*
95200	95800	73892	149226	*rps12*
95400	96000	91422	98286	*ycf2*
95400	96000	73892	103733	*rps12*
95400	96000	73892	149226	*rps12*
95600	96200	91422	98286	*ycf2*
95600	96200	73892	103733	*rps12*
95600	96200	73892	149226	*rps12*
125200	125800	123755	125261	*ndhD*
125200	125800	125385	125631	*psaC*
125200	125800	73892	149226	*rps12*
125400	126000	125385	125631	*psaC*
125400	126000	73892	149226	*rps12*

125600	126200	125385	125631	*psaC*
125600	126200	73892	149226	*rps12*
126000	126600	126263	126569	*ndhE*
126000	126600	73892	149226	*rps12*
126200	126800	126263	126569	*ndhE*
126200	126800	126774	127305	*ndhG*
126200	126800	73892	149226	*rps12*
126400	127000	126263	126569	*ndhE*
126400	127000	126774	127305	*ndhG*
126400	127000	73892	149226	*rps12*
130600	131200	73892	149226	*rps12*
130600	131200	130163	131345	*ndhH*
130800	131400	73892	149226	*rps12*
130800	131400	130163	131345	*ndhH*
131000	131600	131469	131724	*rps15*
131000	131600	73892	149226	*rps12*
131000	131600	130163	131345	*ndhH*
131200	131800	131469	131724	*rps15*
131200	131800	73892	149226	*rps12*
131200	131800	130163	131345	*ndhH*
138000	138600	137955	138027	*trnN-GUU*
138000	138600	73892	149226	*rps12*
138200	138800	138601	138675	*trnR-ACG*
138200	138800	73892	149226	*rps12*
138400	139000	138601	138675	*trnR-ACG*
138400	139000	138938	139059	*rrn5*
138400	139000	73892	149226	*rps12*

Hotspot region:

Start	End	Pi
0	600	0.0315
6200	6800	0.0304
6400	7000	0.0542
6600	7200	0.0544
6800	7400	0.0401
9600	10200	0.0354
9800	10400	0.0355
15800	16400	0.0372
16000	16600	0.0412
16200	16800	0.0456
16400	17000	0.0405
16600	17200	0.036
32200	32800	0.0368
32400	33000	0.044
32600	33200	0.0457

36600	37200	0.0352
36800	37400	0.0377
37000	37600	0.0405
56400	57000	0.0329
56600	57200	0.0322
63200	63800	0.0342
63400	64000	0.0577
63600	64200	0.0808
63800	64400	0.0805
64000	64600	0.0519
64200	64800	0.0471
95200	95800	0.0659
95400	96000	0.0695
95600	96200	0.0391
125200	125800	0.0306
125400	126000	0.038
125600	126200	0.033
126000	126600	0.0303
126200	126800	0.0432
126400	127000	0.0443
130600	131200	0.0411
130800	131400	0.0794
131000	131600	0.0695
131200	131800	0.044
138000	138600	0.033
138200	138800	0.0392
138400	139000	0.0343
166200	166800	0.0384
166400	167000	0.0741
166600	167200	0.0727
166800	167400	0.0307
